# Effects of paracetamol/acetaminophen on the expression of solute carriers (SLCs) in late‐gestation fetal rat brain, choroid plexus and the placenta

**DOI:** 10.1113/EP091442

**Published:** 2023-12-07

**Authors:** Yifan Huang, Fiona Qiu, Katarzyna M. Dziegielewska, Liam M. Koehn, Mark D. Habgood, Norman R. Saunders

**Affiliations:** ^1^ Department of Neuroscience Monash University Melbourne Victoria Australia; ^2^ Drug Delivery, Disposition and Dynamics, Monash Institute of Pharmaceutical Sciences Monash University Parkville Victoria Australia

**Keywords:** brain, choroid plexus, fetus, gene expression, paracetamol, placenta, rat, solute‐linked transporters

## Abstract

Solute carriers (SLCs) regulate transfer of a wide range of molecules across cell membranes using facilitative or secondary active transport. In pregnancy, these transporters, expressed at the placental barrier, are important for delivery of nutrients to the fetus, whilst also limiting entry of potentially harmful substances, such as drugs. In the present study, RNA‐sequencing analysis was used to investigate expression of SLCs in the fetal (embryonic day 19) rat brain, choroid plexus and placenta in untreated control animals and following maternal paracetamol treatment. In the treated group, paracetamol (15 mg/kg) was administered to dams twice daily for 5 days (from embryonic day 15 to 19). In untreated animals, overall expression of SLCs was highest in the placenta. In the paracetamol treatment group, expression of several SLCs was significantly different compared with control animals, with ion, amino acid, neurotransmitter and sugar transporters most affected. The number of SLC transcripts that changed significantly following treatment was the highest in the choroid plexus and lowest in the brain. All SLC transcripts that changed in the placenta following paracetamol treatment were downregulated. These results suggest that administration of paracetamol during pregnancy could potentially disrupt fetal nutrient homeostasis and affect brain development, resulting in major consequences for the neonate and extending into childhood.

## Abstract (highlights)

- **What is the central question of this study?**
  How does maternal paracetamol treatment affect expression of solute carrier (SLC) transporters in the fetal (embryonic day 19) rat brain, choroid plexus and placenta?
- **What is the main finding and its importance?**
  Paracetamol administered to dams at embryonic days 15–19 resulted in changed expression of a large number of SLC transporters in the placenta and the fetal brain and choroid plexus. In the placenta, all SLC transporters that changed expression were downregulated. In the fetal brain and choroid plexus, expression of SLC transporters was both up‐ and downregulated. These findings might have implications for development of fetuses and offspring of mothers who have taken paracetamol during pregnancy.

## 1 INTRODUCTION

Paracetamol (acetaminophen) has previously been thought to be a safe medication when taken at clinical therapeutic doses and has therefore been used widely, even during pregnancy (Australian Medicines Handbook, 2022; Werler et al., 2005; Zafeiri et al., 2021). In addition, it is the only analgesic recommended by the World Health Organization (World Health Organization, 2012) for use in children under the age of 3 months. However, recent retrospective (Brandlistuen et al., 2013; Liew et al., 2014) and prospective (Baker et al., 2020) epidemiological studies, in addition to experimental studies in animal models (Blecharz‐Klin et al., 2017; L. Koehn et al., 2019), have started to question the safety of paracetamol. Human studies have correlated adverse neurodevelopmental effects in children with exposure to paracetamol when the drug was taken by the mother during pregnancy or by the offspring during early development. Paracetamol use has also become a subject of more recent debates in media and medical circles about whether wide availability of this drug should be more regulated (Bauer et al., 2021).

Paracetamol is an analgesic and antipyretic medication widely available over the counter in many countries. Its mechanism of action has still not been elucidated fully, although many have theorized that its analgesic effects could be by acting via CNS‐specific inhibition of the cyclooxygenase (COX) pathway or metabolized to *p*‐aminophenol, which then acts on the endogenous cannabinoid system and transient receptor potential vanilloid 1 (TRPV1) in the midbrain, medulla or spinal dorsal horn (Bertolini et al., 2006; Ghanem et al., 2016; Ohashi et al., 2017).

In adult humans, the clinical therapeutic dose of 15 mg/kg has a plasma half‐life of ∼2 h, and in neonates this is extended to ∼3.5 h (Levy et al., 1975) because sulfation becomes the primary route of metabolic conversion (Levy et al., 1975). In rats, a significantly shorter half‐life of ∼16 min has also been reported after a similar single dose of 15 mg/kg (Galinsky & Levy, 1981).

Paracetamol is metabolized primarily by phase II conjugation in the liver, with 55%–60% glucuronidation via Uridine 5'‐diphospho (UDP)‐glucuronosyltransferase (UGT) enzymes (UGT1A6, UGT1A1, UGT1A9 and UGT2B15) and 20%–30% sulfation via sulfotransferase (SULT) enzymes (SULT1A1, SULT1A3, SULT1A4, SULT2A1 and SULT1E1). Up to 10% turns into the hepatotoxic metabolite *N*‐acetyl‐*p*‐benzoquinone‐imine (NAPQI) via cytochrome p450 enzymes (CYP2E1, CYP3A4, CYP1A2, CYP2D6 and CYP2A6); this is then detoxified via glutathione conjugation by glutathione *S*‐transferase (GST) enzymes (GSTP1, GSTT1 and GSTM1). Around 4% is excreted unchanged in urine (Mazaleuskaya et al., 2015).

In our recent paper (Koehn et al., 2021), the effects of paracetamol exposure on ATP‐binding cassette (ABC) efflux transporters and their associated drug‐metabolizing enzymes in the brain, choroid plexus and placenta at different developmental ages in the rat were explored. The results showed that several of the genes controlling expression of these enzymes, including those that are known to be involved in drug efflux and metabolism, were changed significantly following either single or repeated exposure to paracetamol (15 mg/kg). Most changes in the expression of these genes were already present after a single dose of the drug, and there were minimal changes detected between datasets from animals exposed to an acute or prolonged treatment (Koehn et al., 2021). This indicates that changes in the expression of these transporters occur quickly and are maintained over extended treatment periods, suggesting potential impacts on the functionality of placental exchange and fetal brain homeostasis following paracetamol administration.

Most of the ABC transporters at barrier interfaces use active transport to efflux substrates from cells or cellular compartments. In our previous study, we did not analyse changes in the expression of another major superfamily of transporters, the solute carriers (SLCs). These influx, efflux and bidirectional transporters use facilitated or secondary active transport (Akash et al., 2023; Pizzagalli et al., 2021). The SLCs transport a wide variety of substrates, including ions, amino acids, sugars and drugs, and are becoming recognized as potential therapeutic drug targets for a wide range of diseases (Rask‐Andersen et al., 2013). Therefore, in the present study we describe changes in the expression of transcripts from the SLC superfamily using published datasets from RNA‐sequencing analysis (Koehn et al., 2020, 2021) to investigate whether repeated paracetamol (15 mg/kg, twice daily) administration over 5 days elicited any off‐target transcriptomic changes to SLC transporters in the late‐gestation [embryonic day (E)19] fetal rat brain, choroid plexus and placenta. The results showed that of the three tissues investigated, the most differences in SLC expression between untreated control and paracetamol‐treated groups occurred in the choroid plexus, and overall expression of SLCs in the fetal brain cortex was the lowest. Importantly, all the changes in expression of SLCs detected in the placenta showed downregulation.

## 2 MATERIALS AND METHODS

The RNA‐sequencing datasets were obtained from the previous study (Koehn et al., 2021). The methods and animal procedures are described briefly below.

### 2.1 Ethical approval

No new animals were used in the present study. In the original study from which datasets were obtained (Koehn et al., 2021), all animal experimentation was approved by the University of Melbourne Ethics Committee (Ethics Approval AEC: 10092) and conducted in compliance with the Australian National Health and Research and ARRIVE guidelines 2.0, both the essential 10 and the recommended set. Every effort was made to minimize the distress and suffering of animals, and all animals were handled by experienced Animal House staff and trained experimenters. All experiments were terminated by exsanguination from the right cardiac ventricle of the fetuses and finally the dam. No animal was lost during these experiments.

### 2.2 Experimental animal model

The Sprague–Dawley strain of *Rattus norvegicus* was used in this study. Animals were supplied by the University of Melbourne Biological Research Facility. They were housed in groups of one or two dams (25 cm × 35 cm × 25 cm cages on Breeders Choice paper bedding, made from 99% recycled paper and biodegradable, with no added chemicals), on a 12 h–12 h light–dark cycle with ad libitum access to food (dry pellets of a fixed formulation for rats; Speciality Feeds, Western Australia) and water.

The age group investigated (at experiment completion) was primigravida time‐mated pregnant females at E19. E0 was the day the vaginal plug was found. This age was chosen because E19 is a stage of development when the placenta is still present and the size of the choroid plexus tissue is such that required pooling was minimal. At E19 it is also possible to obtain adequate volumes of cerebrospinal fluid (CSF), which was important for associated drug measurement and permeability experiments that were published in parallel to RNA‐sequencing (Kohen et al., 2019, 2020). At completion of experimentation, all E19 pregnant dams weighed between 350 and 400 g.

Experiments were mostly conducted during the mornings. When possible, similar numbers of male and female fetuses and pups were used, especially when samples had to be pooled. Experimental blinding was also applied and experimenter bias minimized when possible as follows: (1) animals were allocated to individual experiments by the Animal House staff, who were not aware of the experimental protocol; and (2) individual pups were processed by two people, and in most cases, samples that required further analysis were also extracted and annotated by two people.

### 2.3 Drug treatment and anaesthesia

The control animals were untreated (not injected). In this group, fetal samples (see ‘Sample collection’ below) from four dams were collected immediately post mortem (anaesthetic overdose of 25% w/v urethane, Sigma, and exsanguination). In the paracetamol‐treated pregnant animals, a clinical dose of paracetamol (15 mg/kg; Australian Medicines Handbook, 2022) was administered to seven dams via i.p. injection twice daily for 4 days starting at E15, with a final i.v. injection on the fifth day at E19 to allow for complementary drug measurement and permeability experiments in the same animals (Koehn et al., 2019, Koehn et al., 2021). This dosage schedule achieved a clinically relevant plasma concentration of paracetamol, as confirmed by ultra‐high performance liquid‐chromatography‐tandem mass spectrometry (Koehn et al., 2021).

On the day of tissue collection, pregnant animals were terminally anaesthetized with i.p. urethane injection (25% w/v urethane; Sigma; 1 mL/100 g body weight). Once deep anaesthesia was achieved, evidenced by absence of the pedal withdrawal reflex as required by the National Health & Medical Research Council guidelines (2008), animals were placed on a 39°C heating pad in the supine position, and an endotracheal catheter was inserted to maintain a clear airway. Catheters were also inserted into the femoral vein and artery for the final i.v. drug injection and sampling. The catheter was flushed with 0.5 mL of heparinized saline (Hospira, 5000 units/mL) after drug administration. Uterine horns were exteriorized, and fetuses were quickly collected serially, starting from 30 min after the final maternal injection. The viability of each fetus was assessed at the time of collection by observing the colour of the umbilical vessels (Koehn et al., 2019, Koehn et al., 2021).

### 2.4 Sample collection

All surgical instruments were cleaned with RNAaseZAP (Invitrogen) to destroy any RNases before sample collection. For control and paracetamol‐treated animals, four biological replicates were collected for brain tissue from the parietal cortex and lateral ventricular choroid plexuses (Koehn et al., 2019, Koehn et al., 2020, 2021). For placentas, four control biological replicates were collected, and for paracetamol‐treated animals 12 samples were collected, as published previously (Koehn et al., 2020, 2021). A cross‐section of the placenta was taken such that tissue from each placental region was present in each sample. A minimum of four fetuses were taken per litter from each dam for this study. The physiological condition of each fetus was confirmed by a visible heartbeat and the difference in colour between umbilical arterial and venous blood. Viable fetuses were selected randomly. These numbers are summarized in Table 1.

**TABLE 1:** Number of pregnant dams used for collection of fetal tissues and number of biological replicates obtained for untreated and paracetamol‐treated groups.

Treatment group	Dams (*n*)	Fetal biological replicates (*n*)		
		Brain	Choroid plexus	Placenta
Untreated control	4	4	4^a^	4
Paracetamol treated	6	4	4^a^	12

*Note*: Tissues were processed for RNA‐sequencing analysis. Numbers are those from Koehn et al. (2020, 2021). The 12 placental samples were collected in groups of four as part of a replication study of possible effects of paracetamol on placental inflammatory gene expression.

^a^ Lateral ventricular choroid plexuses were pooled from three or four fetuses from the same litter. Tissue samples were collected into RNase‐free cryogenic vials and snap frozen using liquid nitrogen before storage at −80°C until use.

### 2.5 RNA extraction

RNA was extracted using commercially available RNeasy Plus Mini Kits (Qiagen) for placenta and RNeasy Plus Micro kits (Qiagen) for choroid plexus and brain according to the manufacturer's specifications and as described previously (Koehn et al., 2020, 2021). All equipment and workspaces were cleaned with 70% ethanol and RNaseZAP (Invitrogen) before extraction.

### 2.6 Analysis of the RNA‐sequencing dataset

The datasets and analytical methods used in the present study were the same as those described by Koehn et al. (2021). Illumina, Next‐generation sequencing was performed by the Australian Genome Research Facility (AGRF, Melbourne). Runs were 100 bp single reads. Raw data (FastQ) were processed using the Galaxy platform and online software packages (Jalili et al., 2020). Default parameters were used unless otherwise specified. Data files were first checked for quality using FastQC (Andrews, 2023; Galaxy v.0.72) and aligned with HISAT2 (Kim et al., 2015; Galaxy v.2.1.0) using rat reference genome (rn6) in the reverse stranded setting; count files were generated using featureCounts (Liao et al., 2014; Galaxy v.1.6.4+galaxy1). Gene differential expression analysis was conducted using three analysis pathways: EdgeR (R. Liu et al., 2015; Robinson et al., 2010; likelihood ratio; Galaxy v.3.24.1+galaxy1), DESeq2 (Love et al., 2014; Galaxy v.2.11.40.6+galaxy1) and limma‐voom (Law et al., 2014; R. Liu et al., 2015; Galaxy v.3.38.3+galaxy3). Gene synonym names were produced via annotatemyIDs (Dunning, 2017; Galaxy v.3.7.0+galaxy2) as described previously (Koehn et al., 2021). Expression levels were considered statistically different in comparisons of interest if present in at least two of the three analysis pathways at a statistical threshold of an adjusted *P* (p‐adj) value < 0.05 (EdgeR, FDR; DESeq2, P‐adj; limma‐voom, adj.P.Val), and counts were ≥1 normalized counts per million (CPM) in at least one comparison group. Functional annotation analysis was performed using the Database for Annotation, Visualization and Integrated Discovery (DAVID; Huang et al., 2009; Sherman et al., 2022).

All estimates of error are standard deviations.

## 3 RESULTS

### 3.1 Expression of SLCs in untreated control E19 brain, choroid plexus and placenta

Transcripts for a total of 430 solute carriers were identified in untreated control brain, choroid plexus and placenta. After filtering by normalized counts per million (CPM, ≥1), 319 remained, which included members from 64 different subfamilies of transporters. Average normalized CPM values for all SLCs present (≥1 CPM) are summarized in Supporting Information Table S1. These transporters include organic anion transporting polypeptides (OATPs) classified in the *SLCO* superfamily, in addition to organic anion (OAT) and organic cation (OCT) transporters, which make up the *SLC22A* superfamily. Generally, OATPs transport hydrophobic organic anions, OATs transport smaller hydrophilic organic anions, and OCTs transport organic cations (Ghersi‐Egea et al., 2018). However, in addition to endogenous substrates, such as hormones and bile salts, these transporters are also known to facilitate the transfer of numerous drugs, as do several other SLCs (Ghersi‐Egea et al., 2018).

**FIGURE 1:**
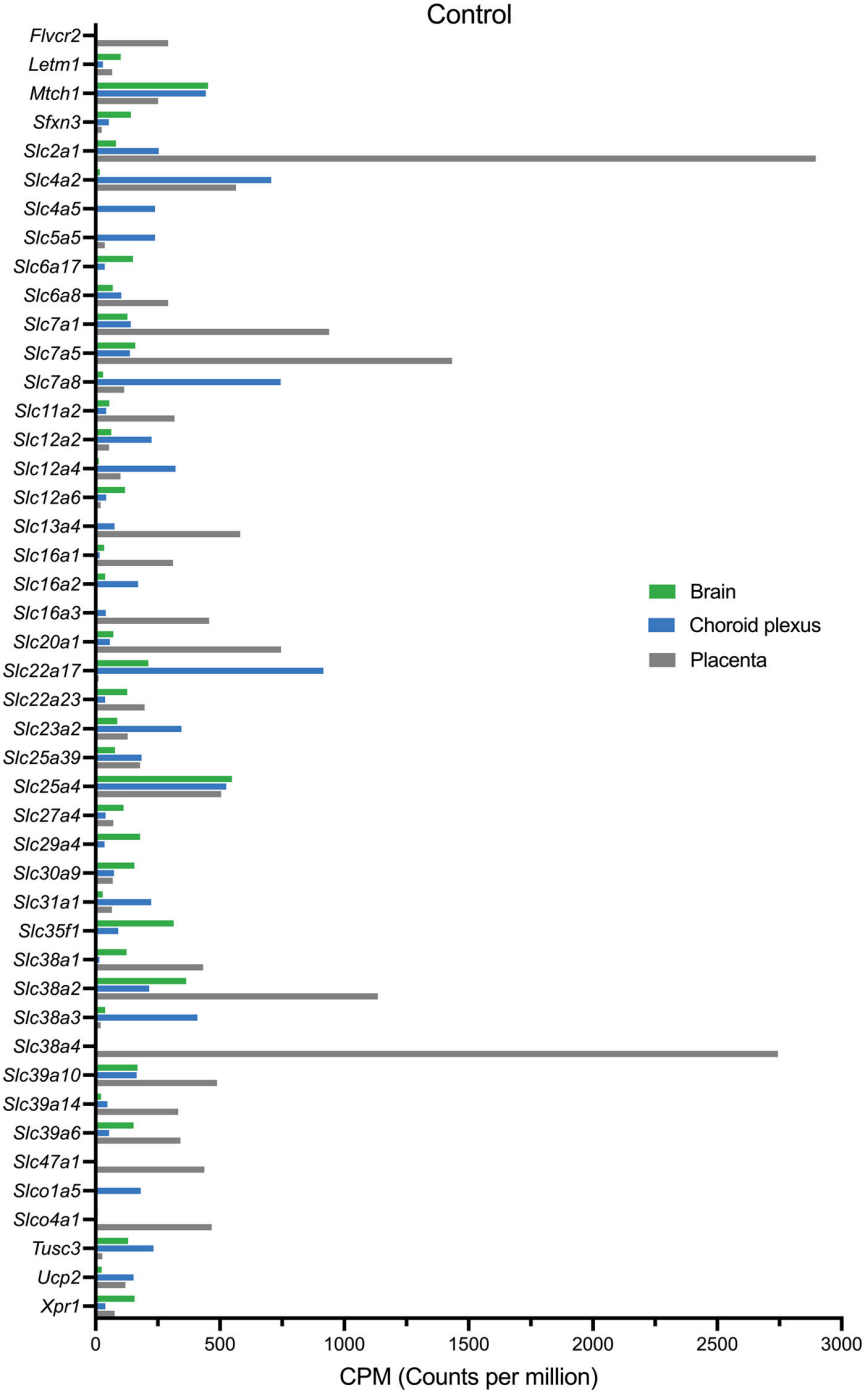
Expression of solute carriers (SLCs) in the untreated control embryonic day 19 rat brain (green, *n =* 4), choroid plexus (blue, *n =* 4) and placenta (grey, *n =* 4) estimated from RNA‐sequencing using average normalized counts per million (CPM) from EdgeR analysis. Note that only the top 20 expressed SLCs present in each tissue were included in this figure. Counts (CPM) are displayed in Supporting Information Table S1.

The top 20 expressed genes in each untreated control tissue investigated (brain, choroid plexus and placenta) are displayed in Figure 1 and Supporting Information Table S2, totalling 45 SLCs when combined. However, 4 of 20 in the brain, 2 of 20 in the choroid plexus and 2 of 20 in the placenta could not be categorized functionally by the program used (DAVID). By manual identification, these were identified in the brain as *Slc27a4* (fatty acid transporter) and *Xpr1* (phosphate exporter). Zinc transporter *Slc39a10* was not categorized in any of the three tissues. *Tusc3* was not categorized in the fetal brain and choroid plexus, and zinc transporter *Slc39a14* was not categorized in the placenta.

**FIGURE 2:**
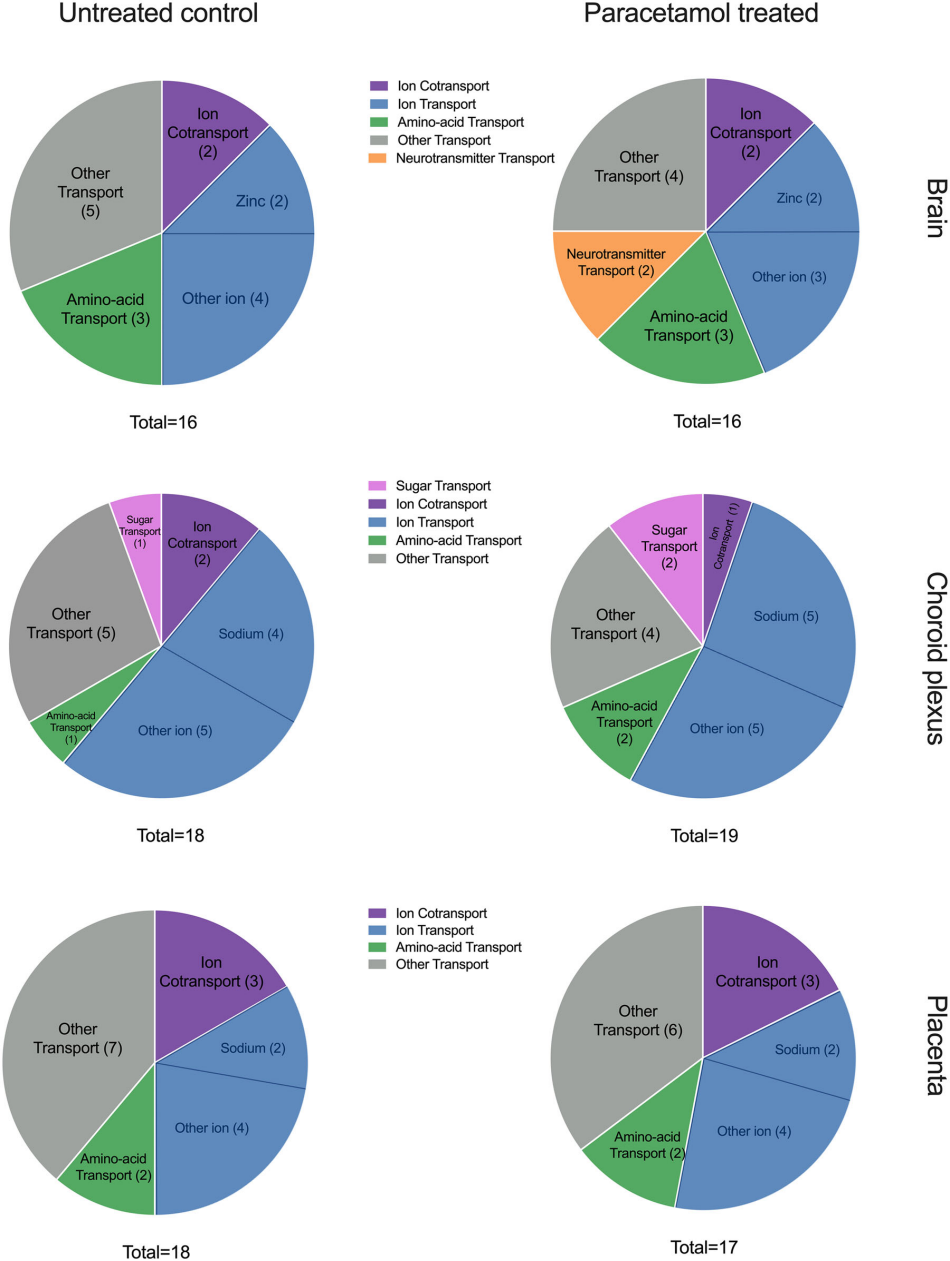
Charts of solute carriers (SLCs) with transporter function in the embryonic day 19 rat brain (*n =* 4), choroid plexus (*n =* 4) and placenta (*n =* 4) in untreated control and paracetamol‐treated rats for the combined top 20 expressed genes in each tissue. Functional annotation of biological processes was conducted using DAVID (UniProtKW). Numbers in parentheses refer to the number of individual transcripts present in each category. Total = number of transcripts included in each analysis; note that this number is <20 because some genes could not be categorized. Ion cotransport refers to transcripts transporting both an ion and another compound displayed in the chart.

The remaining genes were sorted further into their functional categories and are shown in Figure 2. In the brain, 6 of 16 transcripts were categorized as ion transporters, including two zinc transporters (*Slc30a9* and *Slc39a6*), while three SLCs (*Slc7a5*, *Sfxn3* and *Slc7a1*) were amino acid transporters. Nine transcripts out of the total 18 in the fetal choroid plexus were classified as ion transporters, such as sodium transporters (*Slc4a5*, *Slc23a2*, *Slc5a5* and *Slc12a2*), with an additional amino acid transporter, *Slc7a8*. In the placenta, 18 transcripts were identified, including six ion transporters, of which two were sodium transporters (*Slc13a4* and *Slc6a8*). In addition, two amino acid transporters (*Slc7a1* and *Slc7a5*) were also present in the placenta.

#### 3.1.1 Brain

In the untreated control E19 rat brain, transcripts for a total of 244 SLCs were present. Five SLCs were expressed at >200 CPM [*Slc25a4* (548 ± 44 CPM), *Mtch1* (452 ± 26 CPM), *Slc38a2* (364 ± 24 CPM), *Slc35f1* (314 ± 31 CPM) and *Slc22a17* (212 ± 21 CPM)], and an additional 15 transcripts were expressed at >100 CPM, including *Slc29a4* (179 ± 18 CPM), *Slc39a10* (168 ± 15 CPM) and *Slc7a5* (159 ± 21 CPM; Supporting Information Table S1).

#### 3.1.2 Choroid plexus

In the untreated control rat choroid plexus, there was a total of 273 transcripts for SLCs present, 15 of which were expressed at >200 CPM, including *Slc22a17* (916 ± 225 CPM), *Slc7a8* (744 ± 294 CPM) and *Slc4a2* (706 ± 204 CPM). Another 17 transcripts were expressed at >100 CPM, including *Slc25a39* (185 ± 76 CPM), *Slco1a5* (181 ± 90 CPM) and *Slc16a2* (171 ± 96 CPM; Supporting Information Table S1).

#### 3.1.3 Placenta

In the E19 placenta, transcripts for 273 SLCs were present, with four expressed at >1000 CPM. *Slc2a1* was the most highly expressed at 2896 ± 392 CPM, followed by *Slc38a4* (2744 ± 436 CPM), *Slc7a5* (1433 ± 327 CPM) and *Slc38a2* (1135 ± 158 CPM). A further 48 transporters had an expression of >100 CPM, including OATP *Slco4a1* (467 ± 48 CPM), OCT *Slc22a3* (134 ± 33 CPM) and OATs *Slc22a18* (212 ± 38 CPM) and *Slc22a23* (197 ± 32 CPM; Supporting Information Table S1).

#### 3.1.4 Three‐way comparison between tissue datasets from control untreated animals

**FIGURE 3:**
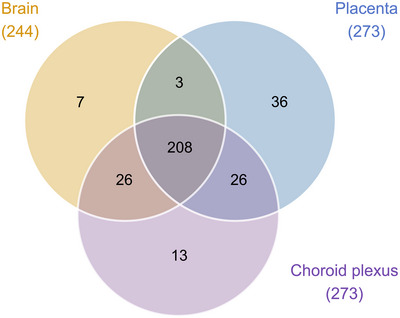
Venn diagram of solute carrier (SLC) transcripts present in embryonic day 19 brain (*n =* 4), choroid plexus (*n =* 4) and placenta (*n =* 4) from untreated control rats identified by RNA‐sequencing analysis. Numbers in paretheses refer to the total number of individual gene transcripts present in each tissue. Data from paracetamol‐treated rats are in Figure 10.

Datasets for the placenta at E19 were compared with datasets for E19 brain and choroid plexus (Figure 3). There were 208 transcripts that were common between all three tissues, including *Slc25a4*, which was highly expressed in all samples: 548 ± 44 CPM in brain, 525 ± 41 CPM in choroid plexus and 505 ± 58 CPM in placenta. The placenta had the highest number of unique transcripts (36), including several highly expressed transcripts, such as *Slc47a1* (437 ± 19 CPM), *Slc52a3* (183 ± 18 CPM), *Slc6a2* (148 ± 8 CPM) and *Slc22a3* (134 ± 33 CPM). Twenty‐six transcripts were shared between the placenta and choroid plexus, including *Slc16a3* and *Slc22a18*, which were highly expressed at >200 CPM in the placenta (456 ± 104 and 212 ± 38 CPM, respectively), in addition to *Slc5a5* and *Slc2a12*, which were expressed at >100 CPM in the choroid plexus (239 ± 99 and 150 ± 61 CPM, respectively). A further 26 transcripts were shared between the fetal brain and choroid plexus, including *Slc29a4* (179 ± 18 CPM in the brain) and *Slc7a10* (117 ± 33 CPM in the choroid plexus), which were expressed at >100 CPM in the brain and choroid plexus, respectively. Only three transcripts were common between the placenta and the brain: *Slc16a14* (3 ± 0.9 and 7 ± 2 CPM, respectively), *Slc9b2* (70 ± 6 and 4 ± 0.6 CPM, respectively) and *Slc26a1* (4 ± 4 and 2 ± 0.3 CPM, respectively).

### 3.2 Expression of SLCs in paracetamol‐treated E19 brain, choroid plexus and placenta

**FIGURE 4:**
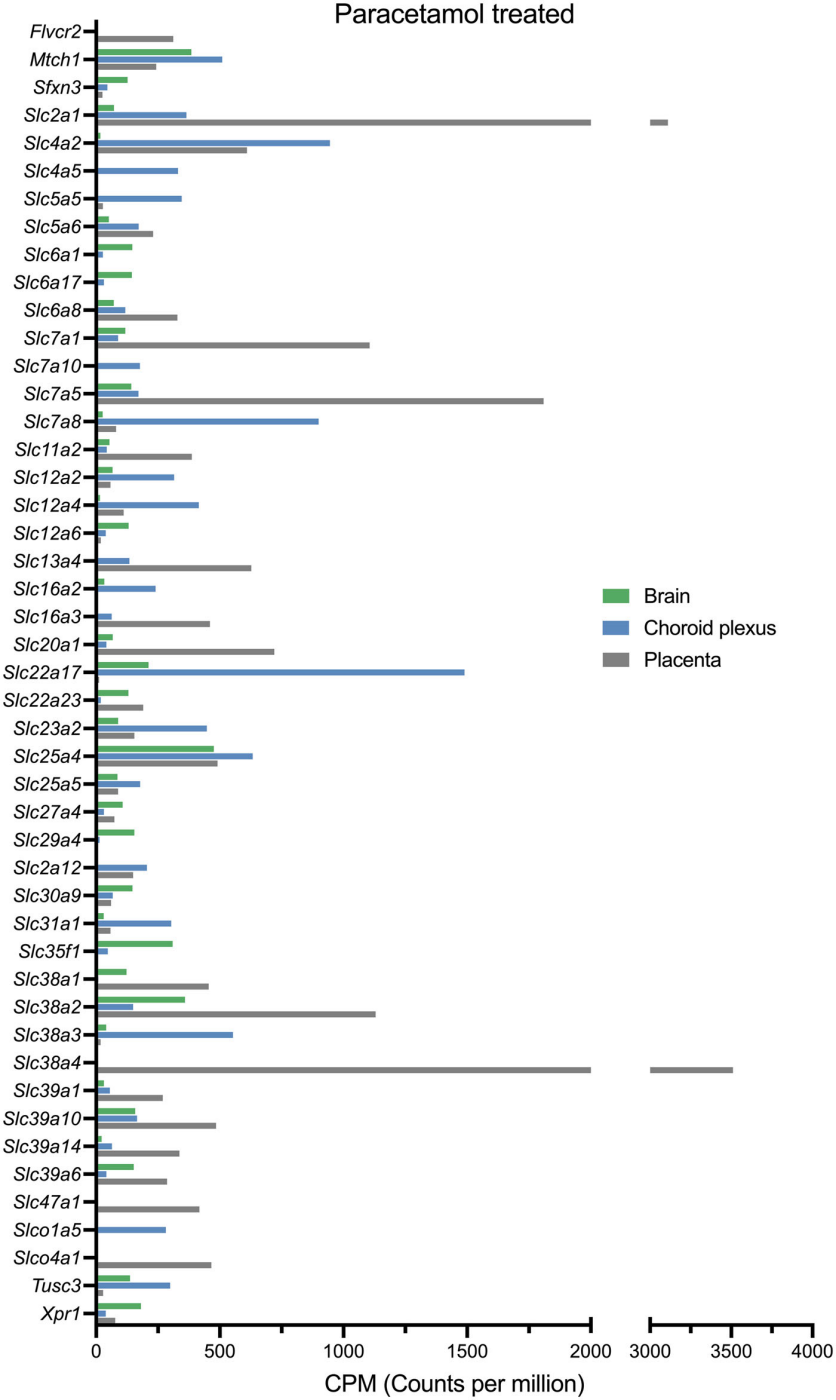
Expression of solute carriers (SLCs) present in the paracetamol‐treated embryonic day 19 rat brain (green, *n =* 4), choroid plexus (blue, *n =* 4) and placenta (grey, *n =* 12) estimated from RNA‐sequencing using average normalized counts per million (CPM) from EdgeR analysis. Note that only the top 20 expressed SLCs present in each tissue were included in this figure. The full list is displayed in Supporting Information Table S1.

Following 5 days of paracetamol treatment, 312 SLCs in total were identified in E19 placenta, brain and choroid plexus. Thirty‐nine of these were highly expressed at >200 CPM (Supporting Information Table S1). The top 20 expressed SLCs in each paracetamol‐treated tissue are displayed in Figure 4 and Supporting Information Table S3, and their functional categories are displayed in Figure 2. In addition to ion and amino acid transporters found in the control animals (Figure 2), an extra sugar transporter [*Slc2a12* (205 ± 19 CPM)] was present in the choroid plexus.

When the top 20 expressed SLCs in all three tissues were combined in the paracetamol‐treated group, a total of 47 unique SLCs were found. As with the untreated controls, some transcripts were uncategorized (4 of 20 in the brain, 1 of 20 in the choroid plexus, and 4 of 20 in the placenta), and the remainder were sorted into their functional categories. In the brain, 5 of 16 transcripts were categorized as ion transporters, including two zinc transporters [*Slc30a9* (147 ± 5 CPM) and *Slc39a6* (152 ± 2 CPM)], three amino acid transporters [*Sfxn3* (127 ± 4 CPM), *Slc7a1* (118 ± 5 CPM) and *Slc7a5* (142 ± 16 CPM)] and two neurotransmitter transporters [*Slc6a1* (146 ± 46 CPM) and *Slc6a17* (144 ± 15 CPM)]. In the choroid plexus, 10 of 19 were SLCs involved in ion transport, such as sodium transporters *Slc23a2* (447 ± 29 CPM), *Slc5a5* (346 ± 37 CPM) and *Slc4a5* (331 ± 48 CPM). In addition, two amino acid transporters [*Slc7a10* (177 ± 28 CPM) and *Slc7a8* (899 ± 74 CPM)] and two sugar transporters [*Slc2a1* (365 ± 22 CPM) and *Slc2a12* (205 ± 19 CPM)] were also categorized. In the placenta, 6 of 17 SLCs were ion transporters, including two sodium transporters [*Slc13a4* (627 ± 70 CPM) and *Slc6a8* (328 ± 35 CPM)]. The top expressed genes (>1000 CPM) in the placenta also included four amino acid‐transporting SLCs, including *Slc7a1* (1106 ± 228 CPM) and *Slc7a5* (1809 ± 308 CPM), in addition to the ion cotransporters *Slc38a4* (3510 ± 1086 CPM) and *Slc38a2* (1130 ± 159 CPM). Comparison of the top 20 expressed SLCs in the untreated control versus paracetamol‐treated groups for the three tissues identified four genes (*Letm1*, *Slc16a1*, *Slc25a39* and *Ucp2*) as unique to the untreated controls, while six genes (*Slc2a12*, *Slc5a6*, *Slc6a1*, *Slc7a10*, *Slc25a5* and *Slc39a1*) were unique to the paracetamol‐treated group. These genes were all highly expressed and were all within the top 50 most expressed SLCs in the tissues investigated (Supporting Information Table S1).

#### 3.2.1 Brain

Out of the 248 SLCs present in the paracetamol‐treated rat brain, five were expressed at >200 CPM: *Slc25a4* (476 ± 54 CPM), *Mtch1* (385 ± 43 CPM), *Slc38a2* (359 ± 30 CPM), *Slc35f1* (309 ± 8 CPM) and *Slc22a17* (212 ± 23 CPM). Another 15 were also expressed at >100 CPM, including *Xpr1* (181 ± 18 CPM), *Slc39a10* (158 ± 5 CPM) and *Slc29a4* (154 ± 7 CPM; Supporting Information Table S1).

##### 3.2.1.1 Comparison between brain datasets from untreated control and treated E19 animals

**FIGURE 5:**
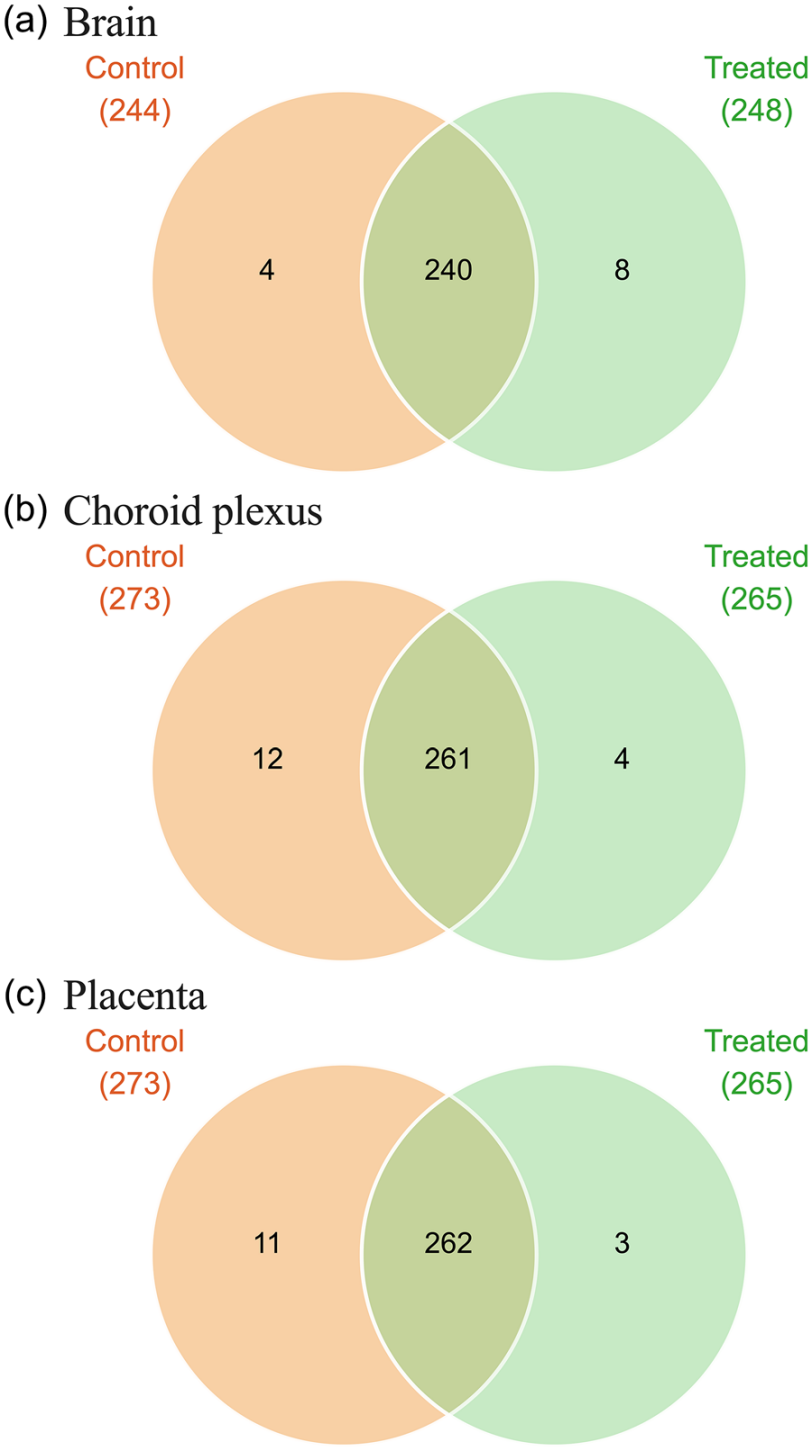
Venn diagram of solute carriers (SLCs) present in the untreated control (orange) and paracetamol‐treated (green) embryonic day 19 rat brain (a), choroid plexus (b) and placenta (c) using RNA‐sequencing datasets. Numbers in paretheses refer to the total number of individual gene transcripts present in each treatment group. There were four biological replicates (*n =* 4) for all groups, except for placenta from the paracetamol‐treated group, for which *n =* 12.

**FIGURE 6:**
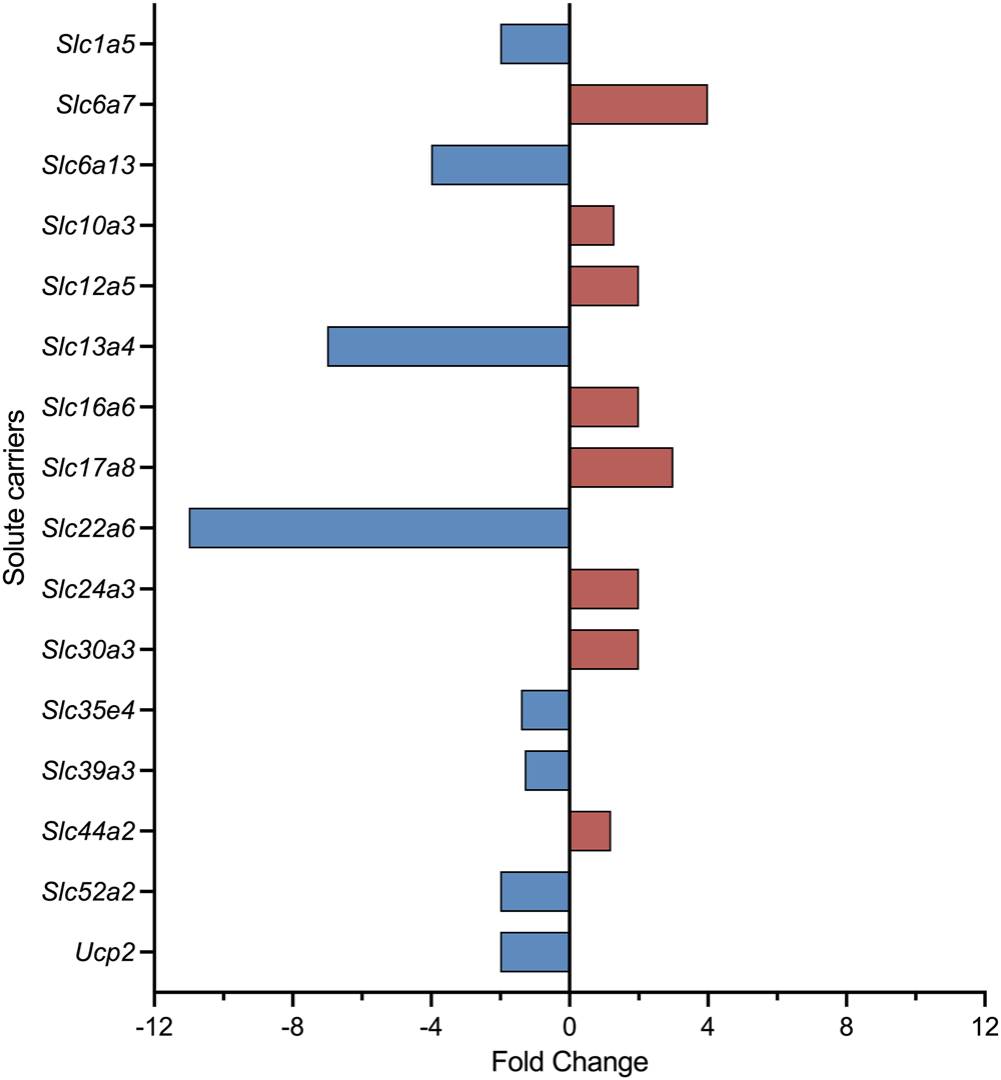
Fold change of solute carriers (SLCs) in the embryonic day 19 rat brain following paracetamol treatment (*n =* 4) compared with untreated control animals (*n =* 4). Those that significantly increased (red) or decreased (blue) their expression are displayed. Expression levels were deemed statistically significant if p‐adj < 0.05 in at least two analysis pathways.

Two hundred and forty SLC transcripts were common between the brain datasets from untreated control and paracetamol‐treated groups (Figure 5a). An additional four transporters were present exclusively in the untreated control group: *Slc22a6* (5 ± 8 CPM), *Slc13a4* (2 ± 3 CPM), *Slc6a13* (1.8 ± 2 CPM) and *Slc12a8* (1.4 ± 0.5 CPM). Comparisons of transcript numbers and fold changes are summarized in Figure 6 and Supporting Information Table S4.

**FIGURE 7:**
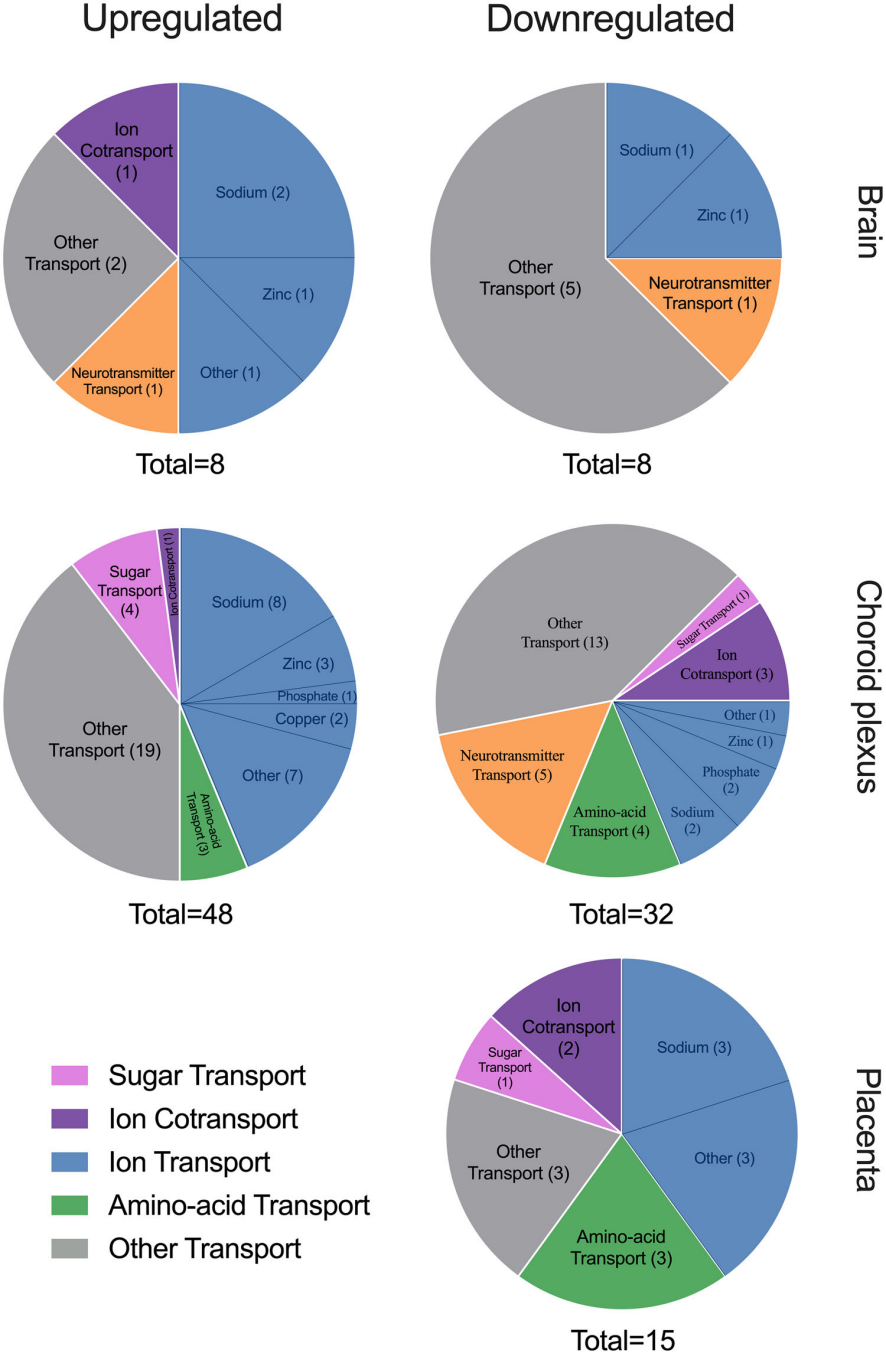
Chart of upregulated and downregulated solute carriers (SLCs) with transporter function in the embryonic day 19 rat brain, choroid plexus and placenta following paracetamol treatment compared with untreated control animals. Functional annotation of biological processes was conducted using DAVID (UniProtKW). Numbers in parentheses refer to the number of individual transcripts present in each category. Total = number of transcripts included in each analysis. Ion cotransport refers to transcripts transporting both an ion and another compound displayed in the chart. There were four biological replicates (*n =* 4) for all groups, except for placenta from the paracetamol‐treated group, for which *n =* 12.

Functional annotation analysis is displayed in Figure 7. Expression of eight transcripts increased significantly following paracetamol treatment. Among these were the neurotransmitter transporter *Slc6a7* (4‐fold, p‐adj = 0.003) along with five ion transporters, such as *Slc17a8* (3‐fold, p‐adj = 0.012) and *Slc30a3* (2‐fold, p‐adj = 0.048). Expression of an additional eight SLCs decreased after paracetamol treatment, such as the organic anion transporter *Slc22a6* (*Oat1*; 11‐fold, p‐adj = 0.012), sodium transporter *Slc13a4* (7‐fold, p‐adj < 0.001) and neurotransmitter transporter *Slc6a13* (4‐fold, p‐adj = 0.014)

#### 3.2.2 Choroid plexus

A total of 264 SLCs were identified in the treated rat choroid plexus at E19. A single transcript, ion transporter *Slc22a17*, had an expression of >1000 CPM (1489 ± 81 CPM). Sixteen others had an expression of >200 CPM, including *Slc4a2* (945 ± 68 CPM), *Slc7a8* (899 ± 74 CPM) and *Slc25a4* (633 ± 41 CPM), and a further 20 transcripts were expressed at >100 CPM, such as *Slc25a5* (178 ± 22 CPM), *Slc7a10* (177 ± 28 CPM) and *Slc5a6* (172 ± 12 CPM; Supporting Information Table S1).

##### 3.2.2.1 Comparison between choroid plexus datasets from untreated control and treated E19 animals

Of the SLCs expressed in the choroid plexus, 261 were present in both the untreated control and paracetamol‐treated groups (Figure 5b). Twelve additional transcripts were present only in the untreated control group, such as *Slc18a2* (14 ± 19 CPM), *Rhag* (2 ± 1.6 CPM) and *Slc18a1* (2 ± 2 CPM), whereas *Slc35g1* (1.4 ± 0.5 CPM), *Slc6a7* (1.4 ± 1.1 CPM), *Slc15a3* (1.2 ± 0.5 CPM) and *Slc5a9* (1.2 ± 0.5 CPM) were present only in the paracetamol‐treated group.

**FIGURE 8:**
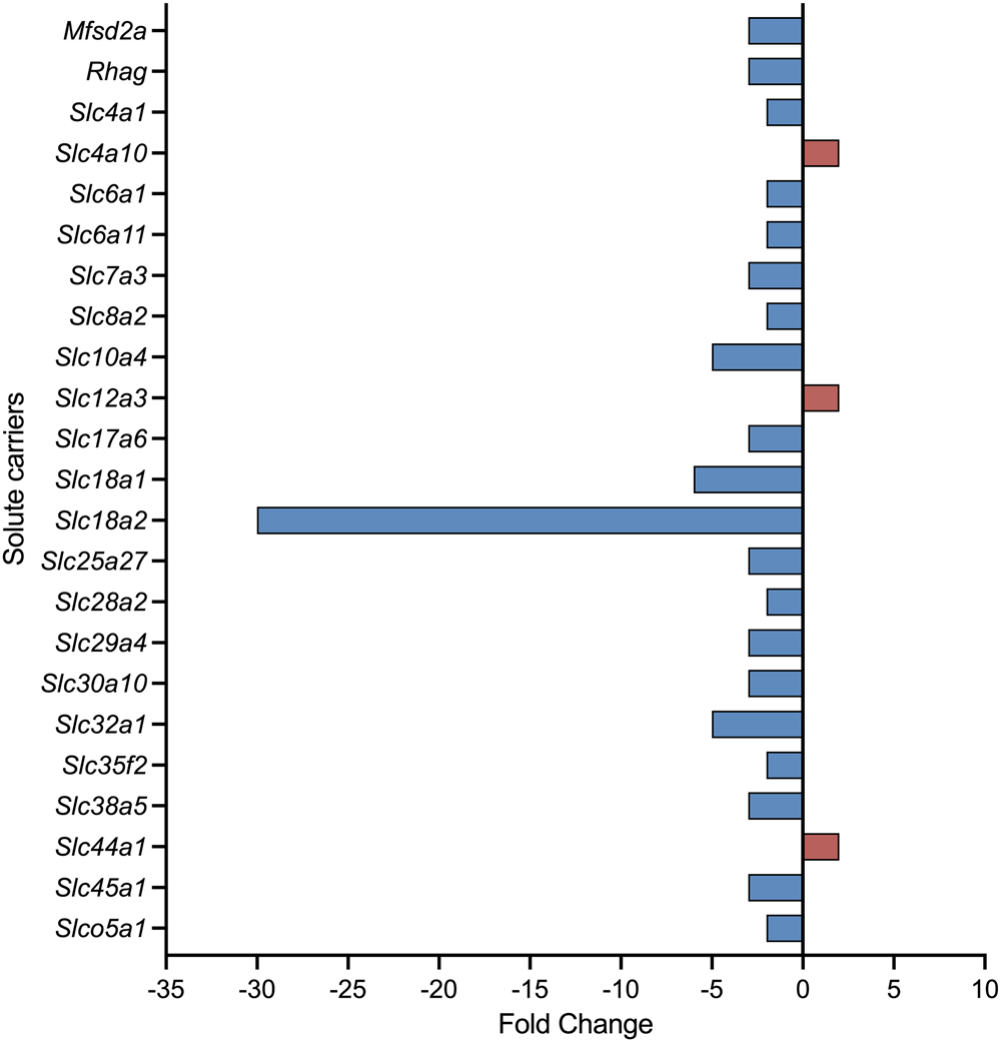
Fold change in expression of solute carriers (SLCs) in the embryonic day 19 rat choroid plexus following paracetamol treatment (*n =* 4) compared with untreated control animals (*n =* 4). Those that significantly increased (red) or decreased (blue) their expression are displayed. Expression levels were deemed statistically significant if p‐adj < 0.05 in at least two analysis pathways. Note that only comparisons with >2‐fold change were included. Almost all these genes were downregulated. The full list is available in Supporting Information Table S5.

Eighty transcripts were changed significantly between the untreated control and paracetamol treatment groups (Supporting Information Table S5). Expression of 21 ion transporters, such as *Slc12a3* (2‐fold, p‐adj = 0.018), *Slc4a10* (2‐fold, p‐adj = 0.020) and *Slc24a4* (1.9‐fold, p‐adj < 0.0001), four amino acid transporters [*Slc3a1* (p‐adj = 0.024), *Slc7a10* (p‐adj = 0.013), *Slc7a4* (p‐adj = 0.007) and *Slc38a3* (p‐adj = 0.035; all <2‐fold change)] and four sugar transporters [*Slc2a1* (p‐adj < 0.001), *Slc35a2* (p‐adj = 0.001), *Slc35a3* (p‐adj = 0.014) and *Slc50a1* (p‐adj = 0.019; all <2‐fold change)] were among 48 SLCs that were increased significantly after paracetamol treatment. Of the 32 transcripts that decreased their expression, nine were ion transporters, such as *Slc10a4* (5‐fold, p‐adj < 0.001), *Slc17a6* (3‐fold, p‐adj < 0.001) and *Slco5a1* (2‐fold, p‐adj = 0.001). The three transcripts that decreased the most following paracetamol treatment were neurotransmitter transporters *Slc18a2* (30‐fold, p‐adj < 0.0001), *Slc18a1* (6‐fold, p‐adj < 0.0001) and *Slc32a1* (5‐fold, p‐adj < 0.001), displayed in Figure 8 and Supporting Information Table S5. Functional annotation analysis is displayed in Figure 7.

#### 3.2.3 Placenta

A total of 265 SLCs were present in the E19 placenta from paracetamol‐treated animals, five of which were expressed at >1000 CPM: *Slc38a4* (3510 ± 1086 CPM), *Slc2a1* (3110 ± 199 CPM), *Slc7a5* (1809 ± 308 CPM), *Slc38a2* (1130 ± 159 CPM) and *Slc7a1* (1106 ± 228 CPM). Another 20 transcripts, including *Slc20a1* (720 ± 104 CPM), *Slc13a4* (627 ± 70 CPM) and *Slc4a2* (610 ± 78 CPM), had an expression of >200 CPM, and an additional 23, such as *Slc22a23* (190 ± 30 CPM), *Slc52a3* (187 ± 28 CPM) and *Slc6a2* (176 ± 19 CPM), were expressed at >100 CPM (Supporting Information Table S1).

##### 3.2.3.1 Comparison between placenta datasets from untreated control and treated E19 animals

Transcripts for 262 SLCs were present in both the untreated control and paracetamol‐treated E19 placenta (Figure 5c). In addition, transcripts for a further 11 SLCs were exclusive to the untreated control group [including *Slc2a2* (25 ± 27 CPM), *Slc13a3* (19 ± 20 CPM) and *Slc7a9* (11 ± 12 CPM)] and for 3 SLCs [*Slc12a3* (1.4 ± 0.7 CPM), *Slc16a11* (1.3 ± 0.4 CPM) and *Slc7a11* (1 ± 0.9 CPM)] that were exclusive to the paracetamol‐treated group.

**FIGURE 9:**
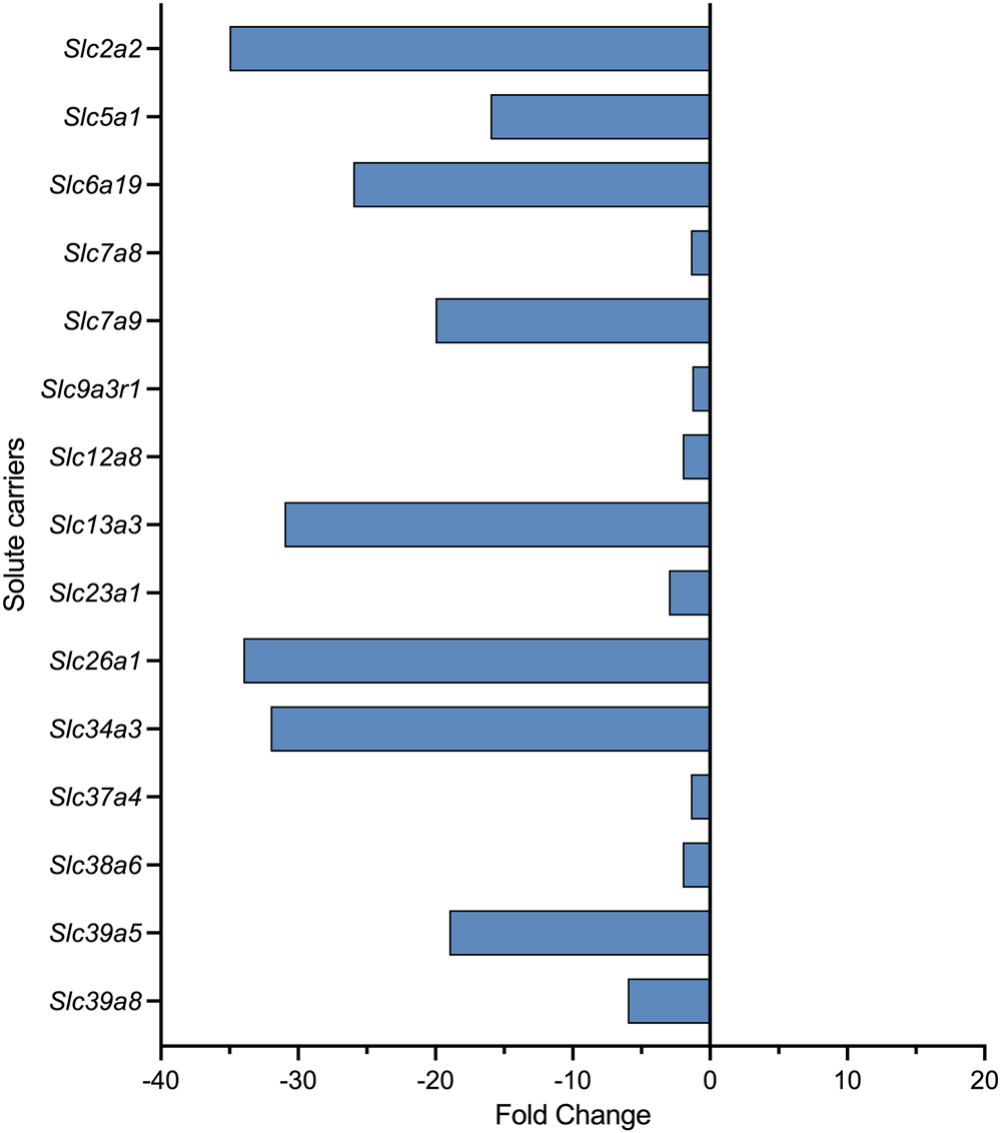
Fold change of solute carriers (SLCs) in the embryonic day 19 rat placenta following paracetamol treatment (*n =* 12) compared with untreated control animals (*n =* 4). Those that significantly decreased (blue) their expression are displayed. Expression levels were deemed statistically significant if p‐adj < 0.05 in at least two analysis pathways. Note that, unlike brain and choroid plexus, no transporters showed significantly increased expression following paracetamol treatment.

Compared with untreated controls, paracetamol treatment decreased expression of 15 SLC transporters, eight of which were ion transporters, including five sodium transporters, such as *Slc34a3* (32‐fold, p‐adj = 0.049), *Slc13a3* (31‐fold, p‐adj < 0.0001) and *Slc5a1* (16‐fold, p‐adj < 0.001). In addition, four amino acid transporters [*Slc6a19* (26‐fold, p‐adj < 0.001), *Slc7a9* (20‐fold, p‐adj < 0.0001), *Slc38a6* (also ion transporter; 1.9‐fold, p‐adj < 0.001) and *Slc7a8* (1.4‐fold, p‐adj = 0.020)], including *Slc2a2* (35‐fold, p‐adj < 0.0001), *Slc26a1* (34‐fold, p‐adj < 0.0001) and *Slc34a3* (32‐fold, p‐adj = 0.049), decreased to very low levels (<1 CPM). There were no SLCs that increased their expression following paracetamol treatment. Transporters with significant decreases are displayed in Figure 9 and Supporting Information Table S6, and functional annotation analysis is illustrated in Figure 7.

#### 3.2.4 Three‐way comparison between datasets from paracetamol‐treated animals

**FIGURE 10:**
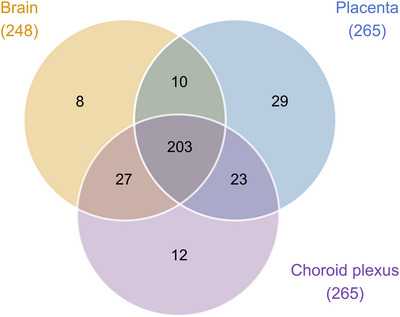
Venn diagram of solute carrier (SLC) transcripts present in embryonic day 19 brain (*n =* 4), choroid plexus (*n =* 4) and placenta (*n =* 12) from paracetamol‐treated rats identified by RNA‐sequencing analysis. Numbers in parentheses refer to the total number of individual gene transcripts present in each tissue. Control data are in Figure 3.

Datasets from the placenta, brain and choroid plexus from paracetamol‐treated animals were compared (Figure 10). The majority of expressed SLCs (203 of 312) were shared between the three tissues, with the ADP/ATP transporter *Slc25a4* being one of the most highly expressed (476 ± 54 CPM in brain, 633 ± 41 CPM in choroid plexus, and 491 ± 37 CPM in placenta).

The placenta had the least in common with either the brain or the choroid plexus, with 29 unique SLCs identified. These included *Slc47a1* (417 ± 43 CPM), *Slc52a3* (187 ± 28 CPM), *Slc6a2* (176 ± 19 CPM) and *Slc22a3* (147 ± 23 CPM), all of which were expressed at >100 CPM. In addition, 12 transcripts were present only in the choroid plexus, such as *Slc4a5* (331 ± 48 CPM) and *Slco1a5* (282 ± 43 CPM). Eight were found only in the E19 brain, but none of these exceeded 15 CPM. Similar to the comparison between datasets from untreated controls (Figure 3), more transcripts were shared between the placenta and choroid plexus (23) than between the placenta and the brain (10). Of the 23 transcripts shared between the placenta and the choroid plexus, 17 were also found in both untreated control tissues (Figures 3 and 10). The remaining six transcripts included sodium/sulphate cotransporter *Slc13a4*, which was expressed at 627 ± 70 CPM and 134 ± 28 CPM in the placenta and choroid plexus, respectively. In addition to *Slc16a14* and *Slc9b2*, which were also shared between the placenta and brain in the untreated control animals, the highly expressed organic anion transporter *Slco4a1* (466 ± 57 CPM and 8 ± 2 CPM in the placenta and brain, respectively) and seven others were also common to both treated tissues. The brain and choroid plexus were the most alike, with 27 shared transcripts, 23 of which were also common to these tissues in the untreated control group, with the final four (*Slc6a7*, *Slc13a3*, *Slc17a8* and *Slc25a3*) exhibiting relatively low expression (<15 CPM) in all tissues.

## 4 DISCUSSION

In the present study, the transcriptomic profiles of SLCs in the brain, choroid plexus and placental tissue in the late‐gestation (E19) rat were analysed in both untreated control and paracetamol‐treated animals. In all three tissues, several SLCs were expressed at a relatively high level in the untreated controls. Following paracetamol treatment, the same number of SLCs was highly expressed in the fetal brain and placenta, but in the choroid plexus an additional two highly expressed transcripts were identified. This generally high expression of SLCs is in contrast to our previously described expression profiles for ABC efflux transporters in the same tissues, which demonstrated relatively low transcript numbers, with the exception of *Abcb1b* (Pgp) in the placenta (Koehn et al., 2021).

Before the present study, it appears that little was known about effects of paracetamol intake on SLC expression in any tissue; however, Khan et al. (2011) investigated changes in SLC expression in a colon cell line 30 min after paracetamol exposure. They found that of 132 SLCs identified, 115 were over‐expressed, including amino acid and sugar transporters, while 17 were unchanged, indicating that paracetamol can modulate SLC expression. In the present study, expression of SLCs was investigated in the brain, choroid plexus and placenta, where a multitude of transporters changed their expression. In all tissues, ion transporters consistently made up the largest proportion of categorized SLC transporters that were changed significantly following paracetamol treatment. A majority of these were sodium transporters, such as *Slc10a4* in the choroid plexus and *Slc13a4* in the brain, which were significantly downregulated. However other SLCs transporting zinc, phosphate and copper increased their expression, including *Slc30a3*, *Slc17a6* and *Slc31a2* in the choroid plexus.

The paracetamol signalling pathway is thought to be mediated by activating serotonergic pathways; however, its primary site of action is still not determined but has been suggested to be by inhibition of prostaglandin synthesis or even by influencing the endocannabinoid system, including results suggesting a brain‐specific pathway that prevents prostaglandin synthesis by a paracetamol metabolite (Saliba et al., 2017). A direct link between SLC expression, tissue specificity and prostaglandin or COX signalling pathways has not been clearly established. In the present study, the expression of the prostaglandin transporter *Slco2a1* (Nakanishi et al., 2021) did not change significantly after paracetamol exposure in any of the tissues investigated, which suggests that this SLC is not involved.

Many SLCs are known to be involved in drug‐related transport, including the organic anion and cation transporters from the *SLCO* and *SLC22* subfamilies (Ghersi‐Egea et al., 2018). Expression of *Slco1a5* and *Slco1c1* was shown to be elevated in the choroid plexus compared with the brain and increased substantially over the course of development (Kratzer et al., 2013). *Slco1a5*, also known as *Oatp3* or *Slc21a7*, transports a variety of substrates, including hormones and drugs (Nagata et al., 2002; Sykes et al., 2004) at the choroid plexus. *Slco1c1* is a thyroid hormone transporter highly expressed in the choroid plexus (Dahlin et al., 2009) and thought to regulate thyroxine uptake from the blood into the CSF (Abe et al., 1998). Of the Slc22 subfamily, *Slc22a5* and *Slc22a8* were highly expressed in the choroid plexus and have also been reported to increase in expression with age in previous investigations in rats (Choudhuri et al., 2003; Kratzer et al., 2013). An additional ion transporter, *Slc22a17*, was the most highly expressed SLC in the choroid plexus. This transporter has recently been hypothesized to play a role in promoting osmotolerance by upregulating gene expression in response to hyperosmolarity and decreasing in response to inflammation (induced by lipopolysaccharides) in the kidney, in a similar fashion to aquaporin‐2 (Probst et al., 2019). However, its substrates and function in the choroid plexus are currently unknown and require further investigation.

Overall, the expression of SLCs in the fetal brain cortex was lower than in the choroid plexus or placenta. In addition to the ion transporter *Slc22a17* and amino acid transporter *Slc38a2*, as mentioned above, three additional highly expressed genes consisted of transporters with a variety of functions: *Slc25a4* (ANT1) encodes for the mitochondrial ADP/ATP‐gated pore carrier (AAC1; Finsterer & Zarrouk‐Mahjoub, 2018; Klingenberg, 2008); *Mtch1* (PSAP), which is present at the mitochondria, has been found to induce apoptosis (Xu et al., 2002); and *Slc35f1*, whose function is still unknown, but is known to be present in the brain and is a member of the Slc35 nucleotide sugar transporter subfamily (Song, 2013). Altogether, these results from untreated control fetal datasets indicate distinct tissue‐specific SLC profiles.

The function of the choroid plexus is to secrete CSF, which both delivers nutrients and removes waste from the brain (reviewed by Liddelow, 2015). It has also been indicated as an important pathway in both ion transport and neurotransmitter homeostasis in the brain (Damkier et al., 2013; Liddelow, 2015; Nilsson et al., 1992). In the present study, of the three tissues investigated, the most differences between untreated control and paracetamol‐treated groups occurred in the choroid plexus, with 48 SLCs significantly upregulating and 32 downregulating their expression. Of those that were upregulated, around half (22) were ion transporters, including sodium, phosphate, zinc and copper transporters, while the most significant change was a downregulation of the neurotransmitter transporter *Slc18a2*. This SLC is known to transport monoamines, such as serotonin, dopamine and histamine (Eiden & Weihe, 2011), and has been found to upregulate in response to stress (Sabban et al., 2012) and downregulate in chronic drug use related to addiction (reviewed by Chang et al., 2007). Five additional neurotransmitter transporters (*Slc17a6*, *Slc18a1*, *Slc32a1*, *Slc6a1* and *Slc6a11*) were also significantly downregulated, with none being upregulated following paracetamol exposure, potentially resulting in a reduction of neurotransmitter transport into the fetal brain.

In the placenta, four transporters were notably highly expressed, including *Slc2a1*, which encodes the glucose transporter GLUT1. GLUT1 is known to be expressed abundantly in both human (cytotrophoblast and syncytial trophoblast cells) and rodent (junctional and labyrinth zones of the chorioallantoic placenta) placenta and is localized to both the apical and basal syncytial trophoblast layers (Burton & Jauniaux, 2023; Knipp et al., 1999; Walker et al., 2017). Expression of placental GLUT1 increases over gestation (Ericsson et al., 2005) and appears to be positively regulated by extracellular glucose (Gaither et al., 1999). In diabetic pregnancies, increased GLUT1 expression increases delivery of glucose to the fetus, therefore potentially contributing to fetal macrosomia (Gaither et al., 1999). In the brain, it has also been proposed as a target for drug delivery across the blood–brain barrier by conjugating drugs with GLUT1 substrates with minimal side effects. In a study by Bilsky et al. (2000), the GLUT1 substrate d‐glucose was conjugated with analgesic opioid agonists and was successful in increasing permeability into the brain. Another study (Arora et al., 2020) investigated the use of GLUT1 to transport brain‐derived neurotrophic factor (BDNF) into the brain as a potential treatment for Alzheimer's disease. In contrast, the other three highly expressed SLCs (*Slc38a4*, *Slc7a5* and *Slc38a2*) were all amino acid transporters (Bröer, 2014; Gyimesi & Hediger, 2023). *Slc7a5* (LAT1) forms a heterodimer with *Slc3a2* (CD98), functioning as an antiport by transporting intracellular substrates in exchange for large neutral amino acids (Häfliger & Charles, 2019). It is crucial in development because it is involved in the transport of eight of the nine essential amino acids to the brain and across the placenta (reviewed by Scalise et al., 2018). The *Slc38* (SNAT) subfamily of amino acid transporters mediate sodium‐dependent transport of small neutral amino acids and play an important role during pregnancy in transporting these amino acids across the placenta to the developing fetus (Bröer, 2014).

The placenta also showed a great number of differences in the paracetamol‐treated group compared with the untreated control group. This is particularly important owing to its role as the first line of defence of the offspring while still in utero and is also the source of its nutrients from the mother in addition to a means to remove waste from the fetus (Griffiths & Campbell, 2015). A striking finding in the present study is that in the placenta all the significant changes in SLC expression were attributable to downregulation and around half of these decreased to <1 CPM. In addition, the decreased expression of these SLCs in response to paracetamol was placenta specific, with no significant change in expression observed in either the fetal brain or the choroid plexus. These downregulated SLCs included ion, amino acid and sugar transporters among others, potentially resulting in reduced transport of important compounds from the mother that could affect fetal development. A link between paracetamol and pre‐eclampsia has been made based on an association between pre‐eclampsia and maternal salt restriction during pregnancy (Sakuyama et al., 2016). However, rather than being a causal factor, this has been attributed to women with pre‐eclampsia being more likely to take paracetamol to treat symptoms in the third trimester (Sahlman et al., 2019; von Hellens et al., 2021). Further investigations, including prospective studies, will need to be conducted to determine whether this hypothesis is true. Although paracetamol has not been associated with adverse perinatal outcomes, such as low birth weight (de Castro et al., 2022), there have been increasing reports of neurodevelopmental effects in the child, including autism spectrum disorder and attention deficit hyperactivity disorder (Baker et al., 2020; Brandlistuen et al., 2013; Liew et al., 2014). Changes in the amino acid profile, especially for neutral amino acids, have also been linked to the incidence of these neurodevelopmental disorders (Cascio et al., 2020; Randazzo et al., 2023; Smith et al., 2019). Some previous studies have noted a disruption in amino acid transporters (Huseinovic et al., 2018) and blood and brain amino acid concentrations (Blecharz‐Klin et al., 2014) following repeated paracetamol exposure. Blecharz‐Klin et al. (2014) found that levels of neutral amino acids (glutamine, glutamic acid, taurine, alanine and aspartic acid) all decreased in the striatum of adult rat brains when compared with untreated controls. However, a prospective study in pregnancy has reported the opposite effect, finding an increase in the concentration of several amino acids (methionine, serine, glycine and glutamate) in umbilical cord plasma that was correlated with both higher concentrations of paracetamol and the oxidative stress marker, 8‐hydroxy‐deoxyguanosine, in addition to a greater risk of the child developing attention deficit hyperactivity disorder (Anand et al., 2021). In the present study, expression of the amino acid transporters *Slc38a6*, *Slc6a19*, *Slc7a8* and *Slc7a9* was substantially decreased in the placenta following exposure to paracetamol. All these transporters function as influx transporters for neutral amino acids, apart from *Slc7a9*, which mediates both the influx of dibasic amino acids and cystine in addition to the efflux of neutral amino acids (Chillarón et al., 2001). Brent and Fawcett (1998) reported the unexpected finding that in E8.5 or younger rat embryos, ≤95% of the amino acids in embryonic tissues were derived from metabolism of maternal proteins. Later in development, placental transfer of amino acids is a prominent feature of placental function in both humans and rodents (Bröer, 2014; Burton & Jauniaux, 2023; Gyimesi & Hediger, 2023; Walker et al., 2017). The practical significance of the findings of reduced expression of amino acid transporters in late‐gestation placenta reported here might be that ingestion of paracetamol in later pregnancy is more of a problem than at earlier stages.

### 4.1 Study strengths and limitations

Pregnant rats were used for this study because it is possible to carry out well‐controlled experiments using the same protocol involving i.v. or i.p. injection in animals that are reasonably homogeneous. Such studies with radiolabelled drugs with these modes of administration involving pregnant women would be ethically unacceptable. Also, as outlined in the Discussion and Conclusions, there are sufficient similarities between essential biological features of the two species that results from rats are likely to have some relevance for human patients.

In clinical settings, paracetamol is most commonly taken orally; however, it can also be administered i.v. In the present study, i.p. and i.v. injections were used for accurate control of the amount of drug administered. These routes of administration were used in the previous drug permeability studies; therefore, a comparison between drug entry and gene changes can be made. Additionally, it would not have been possible for age‐related studies to apply drug treatment orally in very immature pups, and especially E19 fetuses.

As with any study using an animal model, inherent differences between the animals and humans are to be expected. The rat was chosen in the present study because its placenta falls into the same category as humans: haemochorial‐type placenta where the trophoblast layers are bathed in maternal blood (Furukawa et al., 2019; Plant & Zeleznik, 2014). However, there are some structural differences, because the rat placenta is subclassified as haemotrichorial, with three trophoblastic layers separating the maternal and fetal circulation, whereas in humans there is only a single syncytiotrophoblast layer, placing human placenta in the haemomonochorial category (Furukawa et al., 2019; Knipp et al., 1999; Plant & Zeleznik, 2014). As noted in the paper by Koehn et al. (2020), this might mean that any changes in transfer observed in the rat might be amplified in the human. It is also possible that regulation mechanisms between the two species are not identical in all the tissues analysed, hence fold changes or exact transporters that change expression in response to paracetamol treatment might differ. Therefore, future studies analysing changes to SLC transporters in human placental tissue in response to paracetamol exposure would be beneficial, because such investigations are currently limited. Additionally, postnatal day 4 is a stage of brain development equivalent to that of very premature but viable human infants at 22–24 weeks of gestation (Clancy et al., 2001; Workman et al., 2013). These ages have been used in earlier studies of other drugs, which allows comparisons to be made for different drugs (Koehn et al., 2019, Koehn et al., 2020; Toll et al., 2021; Huang et al., 2023).

Although many changes in solute carrier expression were observed at the transcriptomic level, the protein distribution or functionality of these SLCs was not investigated in the present study. Currently, the link between gene expression and gene‐protein product distribution is not well understood, because upregulated genes do not always reflect an increase in the corresponding protein concentration (Greenbaum et al., 2003; Y. Liu et al., 2016). Therefore, further investigations of SLC transporter protein levels and the functional effect on substrate levels in the fetal brain, such as ions and amino acids, are warranted, such as direct measurement of substrate transport across the placenta, proteomic analysis of the transporters or use of transporter‐specific modulation. These approaches might help to expand our understanding of the transport mechanisms of nutrients and removal of waste products between mother and baby during pregnancy and to elucidate the consequences of restricted nutrient transfer essential to the developing child.

A common concern is that treatment of any kind can cause stress to animals; therefore, the response to the drugs could also be attributable to a stress response. We are aware that in some animal handling facilities animals can be very stressed; however, our animals are handled by very experienced staff, and we strive to limit their stress levels as much as possible. We have compared datasets obtained from our control group with those from our treatment groups for several known stress‐induced genes and some common inflammatory markers. Of 17 transcripts tested, only two were marginally increased in the treatment group (*Nfkb1* by 1.4‐fold and *Hsp90b1* by 1.3‐fold). This information is in Supporting Information Figure S1. In a study by Lien et al. (2020), in which inflammation was induced in pregnant mice using lipopolysaccharide, only one (*Nfkb1*) of the same genes identified in our dataset showed a marginal increase in expression. In addition, we have also carried out similar RNA‐sequencing analysis of the same three tissues after a single i.p. injection of paracetamol (data from Koehn et al., 2020, 2021). The results showed that there was no difference between expression of these transcripts after a single injection and injections over several days. This is similar to our previous comparison between datasets from such treatment groups for ABC efflux transporters and their metabolizing enzymes (Koehn et al., 2020, 2021; data availability indicated below). Thus, using control animals that are not injected appears to be justified; additionally, having datasets for fetal rat brain, choroid plexus and placenta from proper controls provides a general database that can be used by others, regardless of their treatment regimes (Koehn et al., 2020, 2021).

## 5 CONCLUSIONS

Paracetamol is taken by ∼70%–80% of pregnant women in many countries (Werler et al., 2005; Zafeiri et al., 2021), making it one of the most commonly drugs used during pregnancy. However, its entry mechanisms into the fetus, the fetal brain and the consequences for the developing child are not well understood. The present study has shown that exposure to paracetamol at clinically relevant levels substantially reduced the expression of many important nutrient‐ and drug‐transporting SLCs, particularly in the placenta but also in the fetal brain and especially the choroid plexus. This has the potential to result in altered fetal exposure to these key molecules and in changes to their homeostatic levels within the developing brain, especially later in fetal development. Further studies investigating the association between paracetamol exposure and nutrient transport are warranted because the disruption of SLC transporter activity could have major consequences for the developing fetus and child.

## AUTHOR CONTRIBUTIONS

Yifan Huang, Norman R. Saunders, Katarzyna M. Dziegielewska, Mark D. Habgood and Liam M. Koehn conceived and designed the study. Yifan Huang and Liam M. Koehn carried out the animal experiments. Yifan Huang prepared the materials for RNA‐Seq analysis and, together with Liam M. Koehn and Fiona Qiu, analysed all RNA‐Seq data. Yifan Huang and Katarzyna M. Dziegielewska wrote the first draft of the paper. Yifan Huang prepared the figures. All authors contributed to revisions of the first draft. All authors approved the final version of the manuscript and agree to be responsible for all aspects of the work in ensuring that questions related to the accuracy or integrity of any part of the work are appropriately investigated and resolved. All persons designated as authors qualify for authorship, and all those who qualify for authorship are listed.

## CONFLICT OF INTEREST

The authors declare no competing interests.

## Supporting information


**Supporting Information Figure S1**. Expression of inflammatory‐ and stress‐related genes in the embryonic day 19 (E19) placenta in untreated control (grey), acute paracetamol‐treated (blue) or prolonged paracetamol‐treated (green) rats measured by RNA‐sequencing using average normalized counts per million (CPM) from EdgeR analysis. *Transcripts that were significantly increased compared with untreated controls (*P* < 0.05).

**Supplementary Table S1**. Normalized counts per million (CPM) of solute carriers (SLCs) present in the untreated control and paracetamol‐treated fetal (E19) rat brain, choroid plexus and placenta using RNA‐sequencing. Yellow cells indicate >100 CPM. Red cells indicate >200 CPM. Green cells indicate >1000 CPM.

**Supplementary Table S2**. Top 20 expressed solute carriers (SLCs) present in the untreated control fetal (E19) rat brain (A), choroid plexus (B) and placenta (C) using RNA‐sequencing. Values are normalized counts per million (CPM). Yellow cells indicate >100 CPM. Red cells indicate >200 CPM. Green cells indicate >1000 CPM.

**Supplementary Table S3**. Top 20 expressed solute carriers (SLCs) present in the paracetamol‐treated fetal (E19) rat brain (A), choroid plexus (B) and placenta (C) using RNA‐sequencing. Values are normalized counts per million (CPM). Yellow cells indicate >100 CPM. Red cells indicate >200 CPM. Green cells indicate >1000 CPM.

**Supplementary Table S4**. Counts [counts per million (CPM)], fold change and adjusted *P* (p‐adj) values of solute carriers (SLCs) with their RefSeq gene identities that were changed significantly between the untreated control and paracetamol‐treated fetal (E19) rat brain using RNA‐sequencing. Counts and fold change values are from EdgeR analysis; changes were considered significant if p‐adj < 0.05 in at least two analysis methods (EdgeR, Limma or DESeq2).

**Supplementary Table S5**. Counts [counts per million (CPM)], fold change and adjusted *P* (p‐adj) values of solute carriers (SLCs) with their RefSeq gene identities that were changed significantly between the untreated control and paracetamol‐treated fetal (E19) rat choroid plexus using RNA‐sequencing. Counts and fold change values are from EdgeR analysis; changes were considered significant if p‐adj < 0.05 in at least two analysis methods (EdgeR, Limma or DESeq2).

**Supplementary Table S6**. Counts [counts per million (CPM)], fold change and adjusted *P* (p‐adj) values of solute carriers (SLCs) with their RefSeq gene identities that were changed significantly between the untreated control and paracetamol‐treated fetal (E19) rat placenta using RNA‐sequencing. Counts and fold change values are from EdgeR analysis; changes were considered significant if p‐adj < 0.05 in at least two analysis methods (EdgeR, Limma or DESeq2).

## Data Availability

The data that support the findings of this study are openly available in NCBI at https://identifiers.org/ncbi/bioproject:PRJNA633629, accession number: PRJNA633629.
