# Supplementary material for: Effects of paracetamol/acetaminophen on the expression of solute carriers (SLCs) in late‐gestation fetal rat brain, choroid plexus and the placenta
Source: Exp Physiol. 2023 Dec 7;109(3):427–44. doi: 10.1113/EP091442 (PMC10988763; doi:10.1113/EP091442)
Supplement: Supplementary file 1 — Supporting Information Figure S1. Expression of inflammatory‐ and stress‐related genes in the embryonic day 19 (E19) placenta in untreated control (grey), acute paracetamol‐treated (blue) or prolonged paracetamol‐treated (green) rats measured by RNA‐sequencing using average normalized counts per million (CPM) from EdgeR analysis. *Transcripts that were significantly increased compared with untreated controls (P < 0.05). [file EPH-109-427-s003.docx]

**Supporting Information Figure S1.** Expression of inflammatory and stress related genes in the E19 placenta in untreated control (grey), acute paracetamol treated (blue) or prolonged paracetamol treated (green) rats measured by RNA-sequencing using average normalised counts per million (CPM) from EdgeR analysis. *Indicates transcripts that were significantly increased compared to untreated controls (p<0.05).
